# Steady agronomic and genetic interventions are essential for sustaining productivity in intensive rice cropping

**DOI:** 10.1073/pnas.2110807118

**Published:** 2021-11-05

**Authors:** Jagdish K. Ladha, Ando M. Radanielson, Jessica Elaine Rutkoski, Roland J. Buresh, Achim Dobermann, Olivyn Angeles, Irish Lorraine B. Pabuayon, Christian Santos-Medellín, Roberto Fritsche-Neto, Pauline Chivenge, Ajay Kohli

**Affiliations:** ^a^Department of Plant Sciences, University of California, Davis, CA 95616;; ^b^International Rice Research Institute, Los Baños 4031, Philippines;; ^c^Centre for Sustainable Agricultural Systems, University of Southern Queensland, Toowoomba QLD 4350, Australia;; ^d^Department of Crop Sciences, University of Illinois at Urbana–Champaign, Urbana, IL 61801;; ^e^International Fertilizer Association, 75116 Paris, France;; ^f^Department of Plant and Soil Science, Texas Tech University, Lubbock, TX 79410;; ^g^Department of Plant Pathology, University of California, Davis, CA 95616;; ^h^African Plant Nutrition Institute, Benguérir 43150, Morocco

**Keywords:** rice, intensive cropping, sustainability, long-term productivity trends, food security

## Abstract

Intensive systems with two or three rice (*Oryza sativa* L.) crops per year account for about 50% of the harvested area for irrigated rice in Asia. Any reduction in productivity or sustainability of these systems has serious implications for global food security. Rice yield trends in the world’s longest-running long-term continuous cropping experiment (LTCCE) were evaluated to investigate consequences of intensive cropping and to draw lessons for sustaining production in Asia. Annual production was sustained at a steady level over the 50-y period in the LTCCE through continuous adjustment of management practices and regular cultivar replacement. Within each of the three annual cropping seasons (dry, early wet, and late wet), yield decline was observed during the first phase, from 1968 to 1990. Agronomic improvements in 1991 to 1995 helped to reverse this yield decline, but yield increases did not continue thereafter from 1996 to 2017. Regular genetic and agronomic improvements were sufficient to maintain yields at steady levels in dry and early wet seasons despite a reduction in the yield potential due to changing climate. Yield declines resumed in the late wet season. Slower growth in genetic gain after the first 20 y was associated with slower breeding cycle advancement as indicated by pedigree depth. Our findings demonstrate that through adjustment of management practices and regular cultivar replacement, it is possible to sustain a high level of annual production in irrigated systems under a changing climate. However, the system was unable to achieve further increases in yield required to keep pace with the growing global rice demand.

## Significance

Steady agronomic and genetic interventions helped sustain high annual rice production in an intensive irrigated monoculture system under a changing climate. However, the system did not achieve the increases in yield required to keep pace with the growing global demand for rice because annual yield potential was stagnant, and apparent biotic constraints limited yield in the wet season.

Major segments of the growing world population have been fed since the 1970s from yield and production growth of rice (*Oryza sativa* L.) and wheat (*Triticum aestivum* L.). Genetic improvement of these cereals, combined with a suite of improved crop management practices and enabled by various policy interventions, came to be known as the Green Revolution. Continued population and income growth drive increasing demand for rice, not only in Asia but also in Africa and other world regions. This challenges current agricultural interventions and policies to transform agricultural systems beyond the first Green Revolution (1, 2) by achieving increased sustainable rice production per unit of land area through higher yields and more intensive cultivation because new land spaces for rice production are scarce (3).

One of the first achievements of the Green Revolution was the release of IR8 in 1966, the first modern short-statured, stiff-strawed, fertilizer-responsive, and non–photoperiod-sensitive rice cultivar (4). This was followed by the release of numerous other high-yielding cultivars that increasingly provided resistance to multiple diseases and insects and also improved grain quality (5, 6). The short duration and photoperiod insensitivity of these cultivars in combination with efficient mechanization, irrigation, and fertilizer management allowed farmers in tropical Asia to harvest two to three rice crops per year, with an annual production of up to about 15 Mg ⋅ ha^−1^ or more (7, 8). Liberal use of water for irrigation and land preparation, where soil is intensively tilled after flooding (puddling or wet tillage) prior to hand transplanting of seedlings, was key to a rapid turnaround in order to achieve three cropping seasons per year in some areas. Irrigated fields producing two to three crops of rice per year today account for up to 50% of the harvested area for irrigated rice in Asia and are hence vital for food security (9, 10).

Historically, rice in Asia has been a very stable production system for thousands of years. However, with the advent of the Green Revolution, scientists and policy makers became concerned about the sustainability of much more intensive forms of rice cultivation and the associated potential changes in soil, water, and environment. More recently, concerns about climate change have reiterated the need to gain a better understanding of the long-term consequences of rice monoculture. Besides monitoring farm productivity, cropping system performance must be measured in controlled field trials over many seasons to assess potential changes in system sustainability and their underlying causes. Such long-term experiments (LTEs) permit quantification of yield relationships with changes in agronomic practices and climate, while factoring out genetic gains for yield and adaptation to emerging biotic and abiotic constraints.

LTEs provide valuable datasets to draw informed judgments about the biophysical aspects of sustainability and to develop strategies for improving cropping systems. In the 1960s and 1970s, many LTEs were established throughout Asia to study yield trends and other changes in double- and triple-cropping irrigated rice systems (11–15). Among those, the long-term continuous cropping experiment (LTCCE) at the International Rice Research Institute (IRRI; Philippines) is unique in that it has three rice cropping seasons annually and is the longest-running rice experiment in the world. Longer-duration experiments with crops other than rice exist at Rothamsted in the United Kingdom and other locations globally, but unlike rice, these crops are not produced on submerged soils which have drastically different biogeochemistry than upland aerobic soil environments (16).

In the LTCCE, the year is divided into dry season (DS), early wet season (EWS), and late wet season (LWS) crops, with a short 2- to 3-wk fallow period between harvest and planting of the next crop. The first comprehensive analysis (1968 to 1990) of rice yields in the LTCCE reported annual yield declines ranging from 1.4 to 2.0% (11). From 1991 to 1995, increased solar radiation, several fallow periods, optimized rate and timing of N fertilizer applications, and enhanced crop protection succeeded in reversing the yield declines (17). Management improvements and regular replacement of rice cultivars have continued since then in an attempt to maintain and increase yields at high levels against a background of a changing climate.

Since the last yield analysis that ended in 1995, 60 more rice crops were harvested in the LTCCE from 1996 to 2017, resulting in a total of 147 crops grown since 1968. With its intensive cropping, the LTCCE acts as a “living laboratory” for investigating potential future constraints to sustained food security through the analysis of crop yield trends. Even though the LTCCE is a scientific experiment at only one location, the LTCCE uses wet soil tillage for land preparation, irrigation, commercial fertilizer, adapted high-yielding cultivars, and crop protection to control weeds, insect pests, and diseases, which are relevant for 22 million ha of harvested area for rice monoculture with two or three crops per year that are an important pillar of food security in the Asia (9, 10). The dry and wet seasons of the LTCCE also reflect the two contrasting rice-growing seasons of monsoonal Asia in which double- or triple-crop rice monoculture systems reside. Furthermore, the LTCCE provides avenues for developing hypotheses for system improvements. A number of specific factors affecting rice yields in the LTCCE during 2001 to 2015 have recently been reported (18). Here, using the LTCCE data for the entire 50-y period, we aim to analyze and understand rice yield trends in two distinct management periods, including comparing the observed trends with the model-simulated climatic-genetic yield potential (referred to as yield potential). In addition, annual rice production trends over 50 y were analyzed. We also estimated relative contributions of genetic and nongenetic improvements to the yield trends. Since agronomic management changed in 1991 and two rice crops instead of three were grown in some years during 1991 to 1995, two main phases with more stable crop and soil management were considered in trend analysis: phase 1 from 1968 to 1990 and phase 2 from 1996 to 2017 (see *Methods* and *SI Appendix*, Tables S1–S4 and Figs. S1 and S2 for details).

## Results

### Annual Production Trends.

**Fig. 1.**
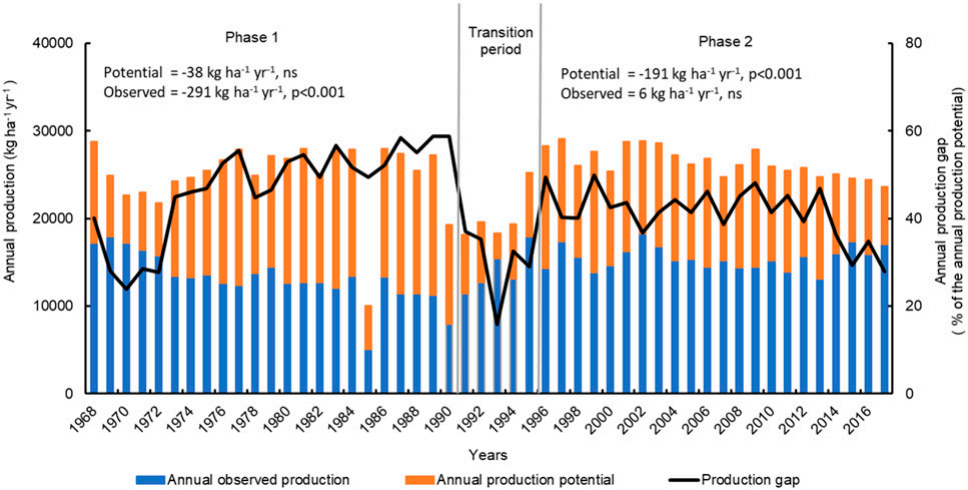
Annual rice production (observed and potential) in the LTCCE over the period from 1968 to 2017 and average production gap from 1968 to 2017. Annual production potential was computed as the sum of climatic yield potential simulated for DS, EWS, and LWS for each year averaged across three cultivars. Observed annual production was computed as the sum of observed yield for DS, EWS, and LWS for each year averaged across the cultivars and the two highest fertilizer-N levels used in the LTCCE. The annual production gap is the difference between the annual production potential and the observed production expressed in % of the annual production potential. Slopes of linear regression of potential and observed annual production with years during the phase 1 period (1968 to 1990) and during phase 2 (1996 to 2017) are presented with level of significance labeled as *** (*P* < 0.001) or ns (nonsignificant). In 1985, the observed yield for LWS was excluded in the total annual production because the crop was damaged by a typhoon.

Over the 50 y from 1968 to 2017, the average annual grain production with full fertilization was 14.4 ± 2.4 Mg ⋅ ha^−1^ with variability among years (Fig. 1). However, the phase-wise time trends differed: a significant rate of decline (−291 kg ⋅ ha^−1^ ⋅ y^−1^; *P* < 0.001) was observed in phase 1 as compared to no significant change in phase 2. On the other hand, the trends of annual production potential were opposite to that of the observed annual production trends. Annual production potential remained unchanged in phase 1 but declined at a rate of −191 kg ⋅ ha^−1^ ⋅ y^−1^ (*P* < 0.001) in phase 2 (Fig. 1). Annual production gap (difference between potential and observed annual production relative to the annual production potential) was higher (46.6%) in phase 1 than in phase 2 (40.8%). The trend of production gap was not significant across 50 y, and interestingly, the annual production gap was lowest during the first 5 y (1968 to 1972) and in most recent years (2015 to 2017) over the 50-y experiment.

### Seasonal Yield Potential Trends.

Yield potential in the DS increased (*P* < 0.05) during phase 1 at a rate of 30.5 kg ⋅ ha^−1^ ⋅ y^−1^ but declined (*P* < 0.001) in phase 2 by 107.6 kg ⋅ ha^−1^ ⋅ y^−1^ (*SI Appendix*, Fig. S3). Likewise, yield potential in the EWS increased (*P* < 0.05) in phase 1 at 59.9 kg ⋅ ha^−1^ ⋅ y^−1^ and decreased significantly in phase 2 by 88.2 kg ⋅ ha^−1^ ⋅ y^−1^, whereas it remained unchanged in the LWS during both periods.

**Fig. 2.**
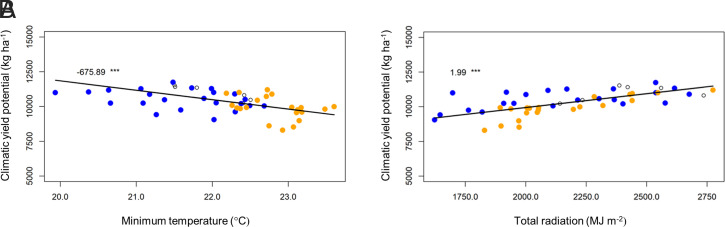
Trends of climatic yield potential with climatic variables namely minimum temperature (*A*) and total radiation (*B*) during DS cropping in the LTCCE. The blue dots are values for phase 1 (1968 to 1990), and the brown dots are values for phase 2 (1996 to 2017). The open circle dots are for the transition period (1991 to 1995). The values are slope of the linear regression of climatic yield potential with variables variation [in kg ⋅ ha^−1^ ⋅°C^−1^ in response to minimum temperature (*A*) and in kg ⋅ ha^−1^ ⋅ MJ ⋅ m^−2^ in responses to total radiation (*B*)] with significance level of *** at *P* < 0.001. The black lines show the linear regression trends.

In the DS, the increase in yield potential in phase 1 was associated with a significant increase in total solar radiation, and the decline in phase 2 was associated with a significant decrease in solar radiation (Fig. 2 and *SI Appendix*, Table S4). An increase in minimum temperature of 0.059 °C ⋅ y^−1^ (*P* < 0.01) probably offset some of the positive influence of solar radiation on yield potential in phase 1 (Fig. 2). In the EWS, the decline of yield potential in phase 2 was associated with a significant decrease of solar radiation and significant increases in both minimum and maximum temperatures (0.015 °C ⋅ y^−1^; *P* < 0.1 and 0.062 °C ⋅ y^−1^; *P* < 0.01, respectively). In the LWS crop, despite the significant increase in maximum temperature and a relatively high minimum temperature of 23.9 °C in phase 2, yield potential showed no significant trends but was very variable from year to year (*SI Appendix*, Table S4 and Fig. S3).

### Observed Seasonal Yield Trends.

Multiple regression models were used to examine the trends of observed yield of rice crops grown under no added fertilizer N (no-NF) and high N input (average of the two highest N rates, referred to as high-NF) treatments across years for each season. Trends in observed yield averaged for all cultivars were determined before separating the variability in yields due to genetic and nongenetic components (*SI Appendix*, Tables S5 and S6).

**Fig. 3.**
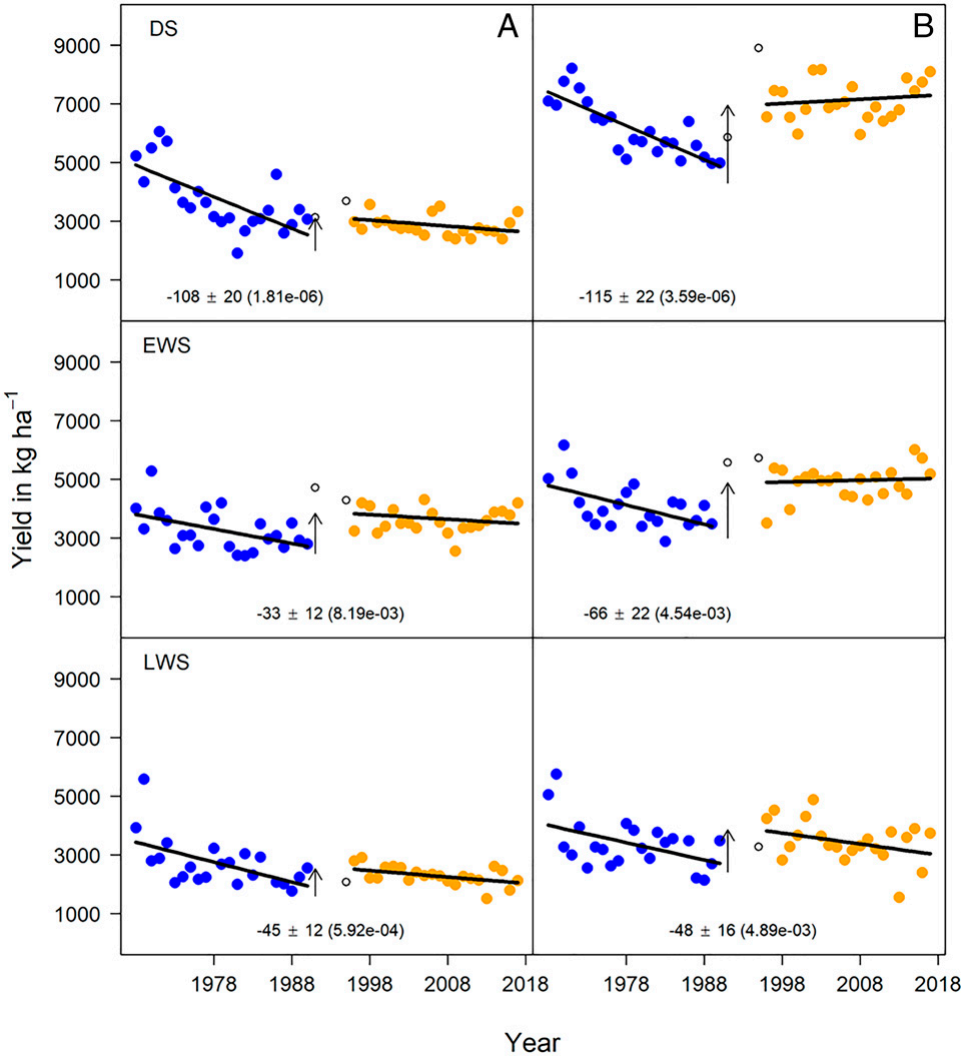
Rice yield trends in the LTCCE by season and time period. Trends are shown separately for each N rate-season combination in separate panels: (*A*) no-NF and (*B*) high-NF. Within a panel, each point is the least-square mean yield of cultivars estimated per trial. Years within phase 1 and phase 2 are blue and orange, respectively. The solid black trend lines show the rate of change in yields per phase. The upward arrows denote a statistically significant increase in yield due to the management changes starting at the beginning of the transition period (1991). Estimates of the rates of change in yields over time (kg ⋅ ha^−1^ ⋅ y^−1^) are written under the trend lines along with SEs (± values) and *P* values, which are in parenthesis. Whenever there was a significant difference in the rate of yield change between phase 1 and 2, both rates are listed under the respective trend line; otherwise, an average rate of change is listed in the center of the panel. When the rate of yield decline is not listed under the trend line panel, it was not significantly different from zero.

With no input of N from fertilizer, rice yields generally declined (Fig. 3*A*). In the DS, a yield decline at a rate of −108 kg ⋅ ha^−1^ ⋅ y^−1^ was observed during 1968 to 1990, but there was no further yield decline in later years. However, in EWS and in LWS, yield declines in no-NF were comparable for both phases at a significant rate of −33 kg ⋅ ha^−1^ ⋅ y^−1^ and −45 kg ⋅ ha^−1^ ⋅ y^−1^, respectively.

In all three seasons, yields declined under high-NF treatment in phase 1. However, except in the LWS, no significant yield trends were observed under high-NF management in phase 2 (Fig. 3*B*). In the DS and EWS crops, the observed yield decreased by about 115 kg ⋅ ha^−1^ ⋅ y^−1^ and 66 kg ⋅ ha^−1^ ⋅ y^−1^, respectively, in phase 1. No significant decline was observed in phase 2. Yield declines in high-NF were consistently observed in the LWS for both phases (−48 kg ⋅ ha^−1^ ⋅ y^−1^). Grain yield responses to fertilizer N were generally higher in phase 2 than in phase 1 (*SI Appendix*, Fig. S4). Although the N use efficiency (NUE) terms showed variable trends, the overall values of agronomic N use efficiency (AEN) were higher in phase 2 than that of phase 1 (*SI Appendix*, Fig. S5). These results indicate that the optimization of agronomic management practices in phase 2 was a major factor that contributed to the sustained observed yields in DS and EWS despite a decline in yield potential (*SI Appendix*, Fig. S3) but not in the LWS crop, which generally had lower yield potential, lower observed yields, and therefore also lower N demand than DS or EWS crops (Fig. 3*B*).

Under high-NF treatment, yield increased significantly between the end of phase 1 and the 5 y of transition (1991 to 1995) in each season (Fig. 3). The yield increase under high-NF was associated with temporarily better climatic conditions (i.e., solar radiation and minimum temperature), fertilizer management optimization (increased rates and split application of N fertilizer), and dry fallow periods resulting from a reduction of cropping seasons from three to two rice cultivars in 1991, 1993, and 1994 (17) (*SI Appendix*, Table S7). Most of the yield increases in DS and EWS were associated with changes in management practices, whereas the yield increase in LWS was primarily associated with changes in climate. For no-NF, the changes in yield between the end of phase 1 and the transition period (1991 to 1995) were not significantly associated with changes in climate (*SI Appendix*, Table S7).

### Nongenetic Yield-Influencing Factors.

The influences of changes in management (fertilizer N rates and splits; sowing date) and climate (solar radiation and minimum daily temperature) on yield were estimated by holding rice cultivar constant. Trends in nongenetic mean yields were estimated from observed data using a combination of mixed statistical models and regression models, which separated the effects of different variables.

#### Nongenetic yield trends under constant management practices.

Under constant cultivars, sowing date, and fertilizer N management, rice yield declined in phase 1 in all seasons for no- and high-NF (Table 1). In phase 2, yield declined in all seasons for no-NF but only in LWS for high-NF. These yield declines can be primarily attributed to factors other than N management, sowing date, and cultivar (e.g., changes in climate, biotic constraints, and the soil resource base).

**Table 1.** Nongenetic yield trends estimated during phase 1 (1968 to 1990) and phase 2 (1996 to 2017), assuming constant cultivars and constant management of fertilizer N and sowing date in the LTCCE

Season*	Fertilizer N^†^	Yield trend (kg ⋅ ha^−1^ ⋅ yr^−1^)^‡^	
		Phase 1	Phase2
DS	No-NF	−47.9 ± 14.8	−47.9 ± 14.8
	High-NF	−105.9 ± 22.3	9.1 ± 21.9
EWS	No-NF	−23.6 ± 15.4	−23.6 ± 15.4
	High-NF	−69.4 ± 24.4	16.3 ± 23.6
LWS	No-NF	−39.9 ± 9.8	−39.9 ± 9.8
	High-NF	−53.0 ± 18.1	−53.0 ± 18.1

*DS, dry season; EWS, early wet season; LWS, late wet season.

^†^No-NF = average of all cultivars with no added N fertilizer and high-NF = average of all cultivars with two highest N fertilizer rates.

^‡^All estimated yield trends were comparable for phase 1 and 2 in LWS. In EWS and in DS, estimated yield trends were comparable under no-NF in both phases while under high-NF trends were not significant in phase 2. ± values are SE.

#### Nongenetic yield trends under constant management practices and climate.

**Fig. 4.**
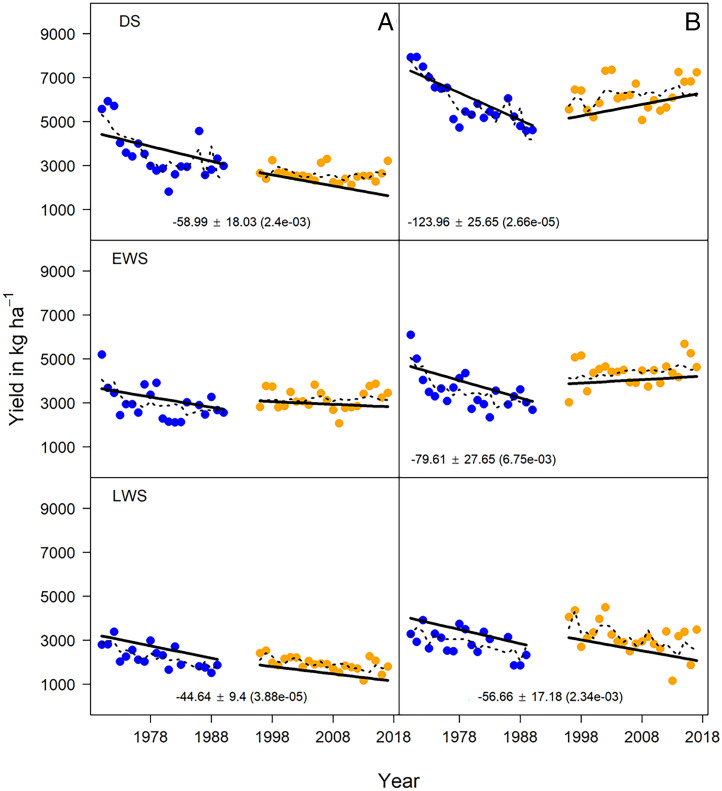
Nongenetic yield trends under constant management practices and climate (N rate, sowing date, cultivar, average minimum temperature, and solar radiation). Trends are shown separately for each N rate-season combination in separate panels: (*A*) no-NF and (*B*) high-NF. Within a panel, each point is the average last-square mean of year estimated using a mixed model analysis combining data across all trials within N rate-season predicted at a constant N rate excluding effect of cultivar. Years within phase 1 and phase 2 are colored in blue and orange, respectively. The solid black trend lines show the rate of change in the predicted yields per phase over time assuming cultivar, N rate, sowing date, minimum temperature, and solar ration are constant. The dotted black lines show the fitted values from the statistical model used to analyze yield trends. Estimates of the rates of change in yields over time (kg ⋅ ha^−1^ ⋅ yr^−1^) are written under the trend lines along with SEs (± values) and *P* values which are in parenthesis. Whenever there was a significant difference in the rate of yield change between phase 1 and 2, both rates are listed under the respective trend line; otherwise, an average rate of change is listed in the center of the panel. When the rate of yield decline is not listed under the trend line panel, it was not significantly different from zero.

With high-NF, yield trends estimated for constant cultivar, sowing date, and fertilizer N management combined with constant climate (i.e., minimum night temperature and seasonal radiation) were negative in all seasons in phase 1 (Fig. 4). For phase 2, DS and EWS yield trends were not significant (Fig. 4*B*). Optimization of fertilizer N management schemes and agronomic practices avoided yield loss from sheath blight (*Rhizoctonia solani*) disease at high N rates and helped maintain the yields during phase 2 in DS and EWS, even though yield potential decreased (*SI Appendix*, Fig. S3) as a result of lower seasonal solar radiation in both seasons and increased daily minimum temperature in EWS (*SI Appendix*, Table S4).

In the LWS, the trends in yield under high-NF treatment were not strongly influenced by climate. The yield declines across phases 1 and 2 with constant climate (Fig. 4*B*) were comparable to yield declines when climate was not held constant (Table 1). The yield declines in LWS were therefore attributed to factors other than changes in N management, sowing date, cultivar, or climate. Biotic constraints such as plant diseases or insect damage likely were contributing factors (*SI Appendix*, Table S8 and Fig. S6).

During DS and LWS, the trends in yield under no-NF treatments were not strongly influenced by climate because the yield declines across phases 1 and 2 with constant climate (Fig. 4*A*) were comparable to yield declines when climate was not held constant (Table 1). Yield declines with no-NF (Fig. 3*A*) in both seasons were therefore attributed to factors other than changes in sowing date, cultivar, and climate. Notably, in the EWS, when controlling for climate, yield decline was not significant in both phases suggesting that in EWS changing climate played a significant role in yield trends (Fig. 4*A*).

In summary, management implemented during DS and EWS in the LTCCE helped compensate the effects of changing climate, leading to steady seasonal yield and annual production over the 50-y period. However, in the LWS, other factors not accounted for in the linear model drove seasonal yield to a continuous decline.

### Genetic Yield-Influencing Factors.

**Fig. 5.**
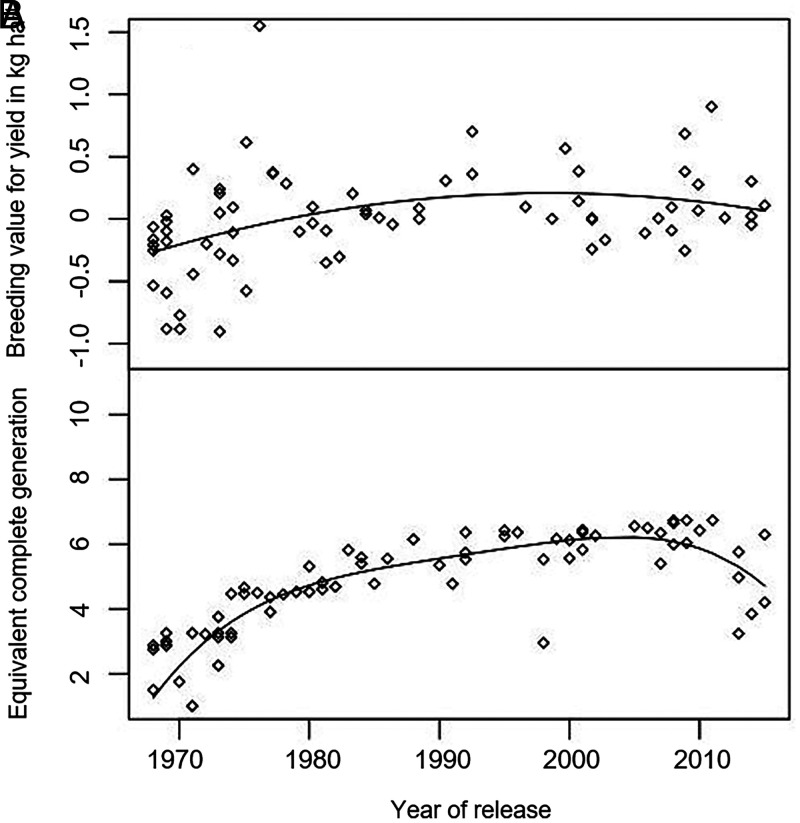
Trends in breeding values and pedigree depth of cultivars across years. (*A*) The relationship in the DS between the breeding values for yield of cultivars, centered at zero, and the year of cultivar introduction in the trial ($\mathrm{Yield}=11.38+1131\;x\;\mathrm{year}-748\;x\;\mathrm{yea}r^{2}$). An increase in breeding values for yield over time can be attributed to genetic improvement due to breeding ($R^{2}=0.16;\;P\;\mathrm{value}\;=\;0.003$). (*B*) The relationship between the pedigree depth (PD) of the cultivars and the year of cultivar introduction in the trial ($PD=4.41+12.30\;x\;\mathrm{year}-7.99\;x\;\mathrm{yea}r^{2}+1.02\;x\;\mathrm{yea}r^{3}-2.03\;x\;\mathrm{yea}r^{4}$). Pedigree depth is measured as the number of the equivalent complete generations which also indicates the number of cycles of breeding that have occurred within a population ($R^{2}=0.78;\;P\;\mathrm{value}\;=\;2.2\text{E}-16$). A cultivar with higher equivalent complete generations is the product of more breeding cycles compared to one with lower equivalent complete generations. In both *A* and *B*, solid black lines depict the best-fitting polynomial curve.

Trends in the genetic component of yield among the rice cultivars used in this experiment were assessed using a mixed model analysis that separated the additive genetic effects from all other nonheritable effects on yield (i.e., management and climate). Genetic yield trends were slightly upward until the late 1990s. In the DS, the genetic yield trend was a second order polynomial with breeding values increasing until about 1997 and then declining slightly thereafter (Fig. 5*A*). Genetic advances in yield occurred more quickly during the first 20 y of the experiment compared to later years. If the rice lines that were entered into this experiment can be considered to be representative of IRRI’s rice breeding germplasm, this trend may provide some insight on genetic gains at Los Baños, Philippines generated over 50 y of rice breeding at IRRI. The lack of increase in genetic effects on yield observed since about 1997 among the rice lines entered into this experiment coincides with a plateauing in the increase in pedigree depth of the cultivars (Fig. 5*B*) as measured by the number of equivalent complete generations (19).

## Discussion

The long-term, intensive rice cropping practiced in the LTCCE provides an accelerated time frame for identifying challenges to sustainable rice farming that might emerge in farmers’ fields. The LTCCE, like nearly all of the 22 million ha of double- or triple-crop rice monoculture systems in Asia (9, 10), used tillage of wet soil for land preparation, irrigation, adapted high-yielding cultivars, fertilizer, and crop protection practices to control weeds, rice pests, and diseases.

Long-term trends suggested that improved agronomic and genetic interventions helped maintain or even restore yields at relatively high levels (Figs. 3 and 4). However, we failed to achieve annual yield increases required to meet future rice demand because the upper yield ceiling (i.e., yield potential), which is determined by the genetics of cultivars and climate, was not higher near the end than at the beginning of the 50-y period (Fig. 1). Depending on future shifts in per capita consumption of rice, annual yield increases of 1.0 to 1.5% would be required to feed Asia without expanding rice cropping to other land areas (3). The LTCCE illustrates the difficulty and complexity of this challenge, even under conditions of applying the best available genetics and agronomic management know-how.

Concerns have often also been raised whether such forms of long-term rice monoculture are tenable in general. However, it is important to keep in mind that the specific hydrological and biological conditions in flooded rice systems provide a very unique and sustainable growing environment that is entirely different from other crops and has sustained Asian civilizations for thousands of years (20). Diversification of triple cropping with nonrice crops can reportedly break pest and disease cycles (21, 22) and provide soil health benefits (23). However, poor soil drainage and risks of submergence at some tropical locations restrict diversification from rice; in such cases, three rice crops per year can provide high annual production potential that is not easily matched by other cropping system choices. Reducing the cropping intensity to just two rice crops per year is another option and would enable longer fallow periods as well as better controls of diseases and insect pests. It would, however, also reduce the annual production potential from ∼25 Mg ⋅ ha^−1^ with three rice crops (Fig. 1) to ∼18 to 20 Mg ⋅ ha^−1^ with optimized planting dates for two rice crops. However, as demonstrated by the LTCCE, triple cropping systems require very careful agronomic management and, if done at larger scale, also good cooperation among farmers to synchronize cropping in order to sustain high levels of productivity (18). Despite higher annual production with three rather than two rice crops, surveys of farmers in the Mekong Delta of Vietnam have indicated that high costs with triple cropping might adversely affect the economic sustainability of triple versus double rice cropping (24, 25).

Our findings also reveal contrasting scenarios for sustaining rice production in dry and wet seasons (EWS and LWS) in the tropics. Yield trends in the DS provide confidence that improved agronomic practices were able to counterbalance negative effect on yield from climate change and climate variability. Improvements in N management through increased rates and changed timing of N applications from 1991 to 1995 and onward helped to increase yield leading to higher response of rice to fertilizer N (including AEN) and stable yield under high-NF treatment in the DS (Fig. 3*B* and *SI Appendix*, Fig. S5). Higher N rates in phase 2 favored sheath blight disease, but changes in agronomic practices controlled sheath blight (*SI Appendix*). Despite yield potential declines in the DS, the maintenance of yield with high-NF in phase 2 (Fig. 3*B*) reflects the general effectiveness of agronomic practices to control biotic constraints such as sheath blight and tungro virus disease in the DS crop (18). However, stagnant observed yields and declining yield potential in DS highlight the critical need for accelerating genetic improvements to increase yield in a changing climate, especially in response to declining solar radiation and increasing minimum (night) temperature (*SI Appendix*, Table S4 and Fig. S7). Analysis of farm-level data from Asia has shown that rice yields at most sites would have grown more rapidly during the high-yielding DS but less rapidly during the low-yielding wet seasons if observed temperature and solar radiation trends at the end of the 20th century had not occurred, with temperature trends being more influential (26). Previous work done at the Los Baños location for the period before 2003 demonstrated that rice yields may decrease by about 10% for every degree of increase in minimum temperature (27, 28). In the LTCCE, throughout the entire 1968 to 2017 period, the climatic yield potential in the DS showed a similar negative relationship of −0.675 Mg ⋅ ha^−1^ per °C increase in minimum temperature (Fig. 2). Clearly, climate change is among the key drivers of the yield potential decline observed in the DS, and the varietal improvement and crop management changes were just enough to keep yields at stable levels. In contrast, in the LWS, yield declines were observed even though the climatic yield potential did not significantly change due to higher minimum temperature, and well-adapted germplasm and good agronomic practices were applied. These yield declines in wet seasons are very concerning because rice is normally the major food crop best adapted to these conditions in Asia. Considering the importance of the wet season for rice production in Asia, one of the major challenges is to close yield and NUE gaps in this season under a variable and changing climate. Furthermore, the failure of agronomic practices within the 1-ha LTCCE area to prevent tungro infection highlights a need for well-synchronized agronomic practices to control biotic stresses at a larger spatial scale, such as synchronized planting (18, 29).

In addition to nongenetic factors, genetic improvement plays a critical role in the future sustainable intensification of rice systems. Our analysis revealed that the genetic improvement among the cultivars in the experiment from 1968 to 2017 was not sufficient to compensate for environmental change and neither did it allow for increasing seasonal yields or annual production of rice. Usually, cultivars tend to reduce their performance over the years for many factors, such as emerging new diseases, breaking down the resistance against diseases, and poor response to new management practices (30). However, our results do not represent this scenario because new improved entries were continuously introduced.

Genetic improvements in yield were particularly slow after the first 20 y of the experiment (Fig. 5), which could be explained by introducing lines into the LTCCE which originated from crosses involving landraces or old cultivars as opposed to advanced breeding materials selected from within the breeding program (31, 32). While crossing with landraces or old cultivars may be useful to introduce novel traits, it is not expected to generate rapid incremental improvements in the average genetic value for yield in a breeding program. Although these crossing decisions were justified for improving tolerance to biotic and abiotic stresses, grain quality, and improved ideotypes, they may have contributed to the lack of continued improvement in genetic yield potential (33, 34).

Changes in the selection criteria or even the germplasm may lead to a small genetic gain, mainly due to the reorganizations of allele frequencies and substitution effects (35). Also, the use of ideotype might have created restrictions in the genetic variability and penalized yield potential (36). We acknowledge that additional studies of genetic trends and pedigree depth with a larger sample are needed to confirm these findings. Nevertheless, the trends in genetic improvement and pedigree depth illustrate why long-term investments in breeding are needed to improve consistently through time.

Large fluctuations in annual production gaps across 50 y confirm the importance of well-tailored agronomic management in sustaining high yield (Fig. 1), but the effectiveness of agronomic management in closing production gaps varied greatly among seasons (*SI Appendix*, Fig. S8). The dynamic improvements in management enabled production gaps near the end of 50 y to match production gap at the start of the experiment in DS and EWS, despite changes in climate. Production gap in LWS, on the other hand, increased during the 50 y, and measured yields near the end of 50 y were only about 50% of yield potential (*SI Appendix*, Fig. S8), which is far below the target of 80% with good management of irrigated rice (37). Yields near the end of 50 y by comparison reached the target of 80% of yield potential in DS but not in EWS. The scope for improving management practices to further close production gaps remains in the wet season, especially for overcoming biotic constraints that are greater in the wet than DS (*SI Appendix*, Fig. S6) and based on simulations tended to increase with time in LWS (*SI Appendix*, Table S8).

## Methods

### Experimental Design and Management of the LTCCE.

The LTCCE began in 1962 at the IRRI experimental farm (named as R.S. Zeigler Experimental Station), Los Baños, Philippines (14°11' N, 121°15' E) and continued with three irrigated rice crops per year from 1968 to 2017. Information on experimental details, soil characteristics, and climatic conditions are provided in *SI Appendix*, Tables S1–S4 and Figs. S1, S2, and S7 and in other published works (11, 17, 18).

The three cropping seasons per year were the DS (January to April), EWS (May to August), and LWS (September to December). The experimental design was a randomized split plot with four N fertilizer rates (no applied N and three rates of increasing N) as main plots, three to six rice cultivars as subplots, and four replications. Each main plot was 24 × 24 m and surrounded by an earthen bund to retain water and fertilizer within the plot. The N rates were higher in the higher-yielding DS than the lower-yielding EWS and LWS (*SI Appendix*, Table S2). During 1968 to 2017, a total of 72 rice cultivars were used (*SI Appendix*, Table S3). One crop was excluded in 1985 due to typhoon damage, and only two crops were grown in 1991, 1993, and 1994.

Agronomic and crop management practices remained relatively constant from 1968 to 1990, but starting in 1991, three changes were introduced. First, N fertilizer management was changed to higher N rates and more times of application (splits). Second, two rice crops instead of three were grown in 1991, 1993, and 1994 to facilitate improvement in the irrigation system (*SI Appendix*). Two crops enabled the soil to dry during the fallow periods between crops and enabled synchronized growth with the two rice crops grown in adjacent fields. Three crops were grown from 1995 to 2017. Third, crop protection was adjusted for increased control of sheath blight disease, golden apply snail, and stemborer (*Scirpophaga* spp., *Chilo* spp.).

Further management changes after 2000 included 1) change in the distribution of fertilizer N among the split applications from 2001 to 2017 to increase the efficiency of N use, 2) decrease in floodwater depth from 2001 to 2017 to reduce canopy humidity and alleviate sheath blight, 3) changes in the growth of rice seedlings and in crop protection starting in 2005 to alleviate rice tungro disease, and 4) changes in crop protection in 2014 to 2017 to alleviate black bug (*Scotinophara coarctata*) in the LWS (*SI Appendix*).

### Measurements.

Crops were manually harvested at physiological maturity close to the ground using sickles, and all above-ground biomass was removed from the plots. Grain yields for each cultivar were measured in a 10-m^2^ harvest area within each subplot. Grain yield was adjusted to 140 g water ⋅ kg^−1^ ⋅ grain.

### Data Analysis.

Total rainfall, total radiation, and average minimum and maximum air temperature for each growing season in each year were obtained daily from data collected at two agrometeorological stations located near the experiment (*SI Appendix*, Table S4 and Fig. S7). Model-simulated climatic rice yield potential was predicted based on actual climatic conditions using the rice model ORYZA version 3, which has been well-calibrated and validated for the conditions at IRRI (*SI Appendix*, Table S9 and Figs. S8 and S9). Each simulation was the average for IR8, IR72, and NSIC Rc158 (IRRI 146), which were three modern benchmark rice cultivars for different periods in the LTCCE. Grain yield response to N was determined based on the partial factor productivity of N and AEN values (*SI Appendix*, Figs. S5 and S10).

Yield trends over the 50 y for each season as well as annual rice production and production gap trends were analyzed (*SI Appendix*, Figs. S9 and S11–S13). Since agronomic management in the LTCCE changed in 1991, annual production and yield trends were analyzed as phase 1 from 1968 to 1990 and as phase 2 from 1996 to 2017. Trends in observed yield for the two highest fertilizer N treatments were not statistically different, and observed yields for these two N rates hence were averaged and referred to as high-NF.

Rice yield trends for no-NF and high-NF averaged for all cultivars were analyzed separately for each season using linear regression and mixed regression models (*SI Appendix*, Table S5 and S6). The total variability in observed yields was decomposed into a genetic component and nongenetic components, which included climate, sowing date, and N management practices. Trends in the nongenetic mean yields were estimated from the measured data using a combination of mixed statistical models and simple regression models, which separated the effects of changes in cultivar from changes in management practices (that included sowing date and fertilizer N rate) and climate (that included solar radiation and minimum daily temperature) (*SI Appendix*, Tables S5 and S6). Trends in yield not due to changes in cultivar, sowing date, N rate, and climate were determined by controlling sowing date, N rate, solar radiation, and minimum temperature through use of these variables as fixed effect covariates in statistical models.

Genetic yield trends are the trends through time in the genetic component of yield, which differs from yield potential (or maximum theoretically attainable yield). Genetic trends in yield were assessed by fitting linear and polynomial regression models to the estimates of the additive genetic values of yield for each cultivar obtained from a mixed model analysis (*SI Appendix*, Tables S5 and S6). The mixed model separated the additive genetic effects from all other effects that are not heritable including management and environment. To better understand trends in additive genetic values, we also examined changes in pedigree depth, which tracks how breeding cycles are progressing. The pedigree depth is the number of generations of crossing that can be traced back until one reaches introduction to the breeding program from external sources (*SI Appendix*, Tables S5 and S6).

## Data Availability

All study data are included in the article and/or *SI Appendix*. Previously published data were used for this work (grain yield data collected from 1968 to 2017 in LTCCE are available in the IRRI Dataverse [https://dataverse.harvard.edu/dataverse/ltcce]).
